# Induction of Monocular Stereopsis by Altering Focus Distance: A Test of Ames’s Hypothesis

**DOI:** 10.1177/2041669516643236

**Published:** 2016-04-28

**Authors:** Dhanraj Vishwanath

**Affiliations:** School of Psychology & Neuroscience, University of St. Andrews, Fife, Scotland, UK

**Keywords:** monocular stereopsis, depth perception, focus cues, equidistance tendency

## Abstract

Viewing a real three-dimensional scene or a stereoscopic image with both eyes generates a vivid phenomenal impression of depth known as stereopsis. Numerous reports have highlighted the fact that an impression of stereopsis can be induced in the absence of binocular disparity. A method claimed by Ames (1925) involved altering accommodative (focus) distance while monocularly viewing a picture. This claim was tested on naïve observers using a method inspired by the observations of Gogel and Ogle on the equidistance tendency. Consistent with Ames’s claim, most observers reported that the focus manipulation induced an impression of stereopsis comparable to that obtained by monocular-aperture viewing.

A significant amount of literature has reported that an impression of stereopsis can be induced in the absence of binocular disparity, for example, by viewing a single picture with one eye through an aperture (Ames, 1925; da Vinci, 1688, cited in Wade, Ono, & Lillakas, 2001; Schlosberg, 1941; Wheatstone, 1838). This claim has been empirically confirmed: Naive observers report the induction of the same qualitative visual attributes under monocular-aperture viewing of single pictures as under stereoscopic perception, including the impression of real negative space and separation, a sense of protrusion (“things stick out towards me”), and an impression of tangibility and realness to objects (Vishwanath & Hibbard, 2013). These findings suggest that the referent of the term “stereopsis” should be the perceptual effect itself (the impression of tangibility and “real separation in depth” between objects; Vishwanath, 2014) rather than a specific condition that induces it (binocular vision). The conditions that yield stereopsis can then be used as a modifier term: e.g., binocular stereopsis, synoptic stereopsis (e.g., Koenderink, van Doorn, & Kappers, 1994) and monocular stereopsis.

Ames (1925) described several methods for inducing monocular stereopsis in single pictures, one of which involved “changing the accommodation of the eyes from that normally required by the distance from which the picture is viewed.” Ames suggested doing this by using concave or convex lenses, and there are suggestions that devices like the Zograscope (Koenderink, Wijntjes, & Kappers, 2013) which also induce the impression of stereopsis rely on a similar idea (Wijntjes, in press). Ames’s proposal has an interesting link to a recent hypothesis regarding the underlying source of stereopsis (Vishwanath, 2011, 2014). This hypothesis claims that the impression of stereopsis is associated with the derivation of the egocentric scale of visual space and that its phenomenal strength depends on the precision with which scaled depth is derived. The hypothesis explains aperture-based monocular stereopsis as deriving from a reattribution of egocentric distance cues required for depth scaling. Under normal binocular viewing of pictures, distance information is ascribed to the visible flat picture surface, leaving pictorial depth unscaled. Monocular-aperture viewing of a picture renders the picture surface invisible. The conjecture claims that residual distance information deriving from focus to the picture surface, along with default distance tendencies, is is misascribed to the pictorial contents causing pictorial depth to be scaled, inducing an impression of stereopsis. The phenomenal strength of this impression is expected to be weaker than binocular stereopsis which derives from high-precision disparity and vergence-based depth scaling (Vishwanath, 2011, 2014).

The link between focus distance and perceived scale has been described by Ogle (1950) and Gogel (1969). Focussing on a close visual target, while covertly attending to another distant target, makes the latter appear closer and smaller (Figure 1(a)). This effect (the “equidistance tendency”) has been explained as the erroneous attribution of distance information deriving from focus to the near target on to the more distant target (Gogel & Teitz, 1977). The effect of an alteration in perceived size in the equidistance tendency is mirrored in monocular-aperture-based stereopsis. Observers report that objects in the picture appear closer and smaller than inferred under normal binocular viewing (Vishwanath & Hibbard, 2013). This is consistent with the conjecture that monocular stereopsis involves a reattribution of distance information (Vishwanath, 2011). Since objects in pictures appear located at some distance beyond the picture surface, attributing distance cues (or tendencies) specifying the distance of the picture surface to pictorial objects should make them appear smaller and closer than under normal viewing. The effect is marked for content depicting distant scenes of large objects, where observers sometimes report a sense of miniaturization (“looks like an architectural model”; Vishwanath & Hibbard, 2013).

**Figure 1.**
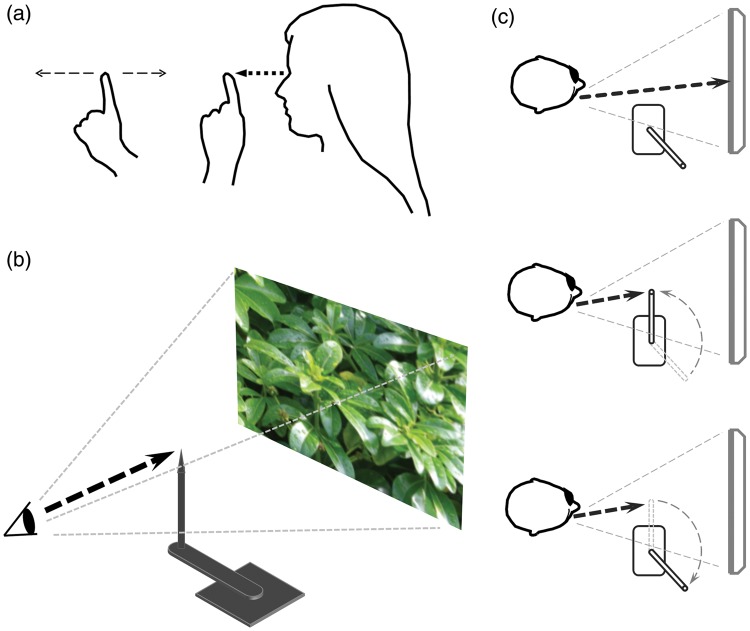
(a) Fixate left index finger monocularly at a distance of 20 to 25 cm while covertly attending to the right index finger as you move it back and forth in depth. The latter appears to shrink and grow in concert with the movement. (b) Stimulus: Photographic image of a 3D scene (31 × 23 cm) displayed 50 cm from the observer; fixation pointer on a rotating arm. (c) Procedure: Subjects viewed the image with their dominant eye for 5 s. The experimenter moved the pointer into the central line of sight and asked subjects to shift their focus onto the pointer while continuing to covertly attend to the image. After 3 s, the pointer was slowly rotated out of the field of view. Subject was instructed to continue to look straight ahead, trying to maintain their previous “focus and attentional state” for an additional 5 s. Observers were asked if they perceived degradation, enhancement, or no difference in depth impression after the focus manipulation.

The conjecture linking the attribution of distance information, depth scaling, and monocular stereopsis, taken together with Ogle’s and Gogel’s observations, suggested a mechanistic basis for Ames’s claim and a way to test it without the use of lenses. Using questionnaires, 10 naïve subjects (19–25 years) were initially screened to establish that they could obtain an impression of monocular stereopsis during monocular-aperture viewing of a single picture (Vishwanath & Hibbard, 2013). They were then tested using the method describe in Figure 1(b) and (c).

They rated any perceived difference in depth impression (operationalized as the “sense of real separation in depth”; Vishwanath, 2014) before and after the focus manipulation based on a scale where 0 indicated no difference and +5 indicated the predefined difference perceived in the reference comparison (monocular aperture vs. binocular viewing of the image).

Two subjects reported that they could not make judgements regarding depth perception because they were unable to see the picture in focus when fixating the pointer and were excluded from the analysis. One subject reported no difference in depth impression. The remaining seven all reported obtaining a heightened impression of depth with the focus manipulation compared to normal monocular viewing, that was qualitatively the same as observed under monocular-aperture viewing (χ^2^ (2,8) = 10.92, *p* = .004). Three subjects reported, spontaneously, that the effect was stronger than monocular-aperture viewing. The average depth-impression difference ratings comparing the focus manipulation to monocular viewing were consistent with ratings obtained in a previous study comparing monocular aperture to monocular viewing (Figure 2).

**Figure 2.**
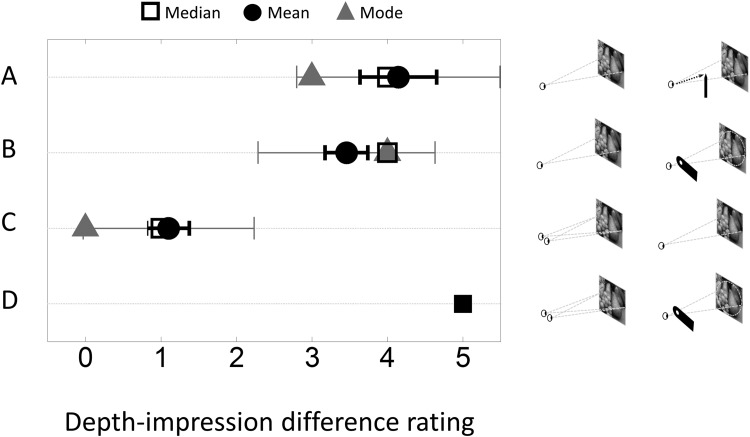
Depth-impression difference ratings. (a) Focus manipulation versus monocular viewing of the image. (b) Monocular aperture versus monocular viewing (data from Vishwanath & Hibbard, 2013). (c) Monocular versus binocular viewing (data from Vishwanath & Hibbard, 2013). (d) The reference comparison (monocular aperture versus binocular viewing) with a predefined value of 5 units. SEMs shown in black; SDs in gray.

Informal testing of a large group of vision researchers (Vision Sciences Society Demo Night) found a similar majority of observers obtaining the effect, though older observers with advanced presbyopia found it difficult to achieve simultaneous focus on the target and picture. These findings confirm the anecdotal claim made by Ames and they provide support for the idea linking monocular stereopsis and reattribution of distance information, though the specific mechanisms underlying this effect remains to be established.
